# Hospital‐Admitted Injection‐Related Infections Among Incarcerated People Who Inject Drugs in Australia: A Retrospective Cohort Study

**DOI:** 10.5694/mja2.70224

**Published:** 2026-06-14

**Authors:** Andrew Palmer, Matthew Carter, Jeremy Yeo, Cecilia Shim, Jason Connor, Jeremy Hayllar, Gerald Holtmann, Naomi Moy, Elliott G. Playford, Naomi Runnegar, Paul J. Clark

**Affiliations:** ^1^ Gastroenterology and Hepatology Princess Alexandra Hospital Woolloongabba Queensland Australia; ^2^ Alcohol and Drug Assessment Unit Princess Alexandra Hospital Woolloongabba Queensland Australia; ^3^ The University of Queensland Herston Queensland Australia; ^4^ National Centre for Youth Substance Use Research The University of Queensland Brisbane Queensland Australia; ^5^ Metro North Mental Health, Alcohol and Drug Service Brisbane Queensland Australia; ^6^ Infection Management Services Princess Alexandra Hospital Brisbane Queensland Australia

**Keywords:** bacterial infections, cost of illness, hepatitis C, mycobacterium, prevention and control, prison, public health, public policy, social justice, substance abuse

## Abstract

**Objectives:**

To characterise the clinical, microbiological and economic burden of hospital‐admitted, injection‐related infections among incarcerated people who inject drugs.

**Study Type:**

Retrospective observational cohort study.

**Setting:**

Secure unit of the Princess Alexandra Hospital, Brisbane, Australia.

**Participants:**

Adults incarcerated in Queensland prisons who were admitted to hospital with an injection‐related infection between 1 July 2019 and 30 June 2023.

**Main Outcome Measures:**

Types of injection‐related infection, microbiological findings, requirement for surgical or radiological source control, hospital length of stay and inpatient healthcare costs.

**Results:**

There were 321 hospital admissions for injection‐related infection among 265 patients, accounting for 282 unique infections. Most patients were male (241; 90.9%), with a mean age of 33 years (standard deviation [SD], 7.4 years), and 76 (28.7%) identified as First Nations. The most frequent infections were soft tissue infections (77/282; 27.3%), acute hepatitis C (64/282; 22.7%) and cellulitis (43/282; 15.2%). Surgical or radiological source control was required in 95 infections (34.0%), and infectious diseases consultation occurred in 130 infections (46.1%). Among 39 true‐positive blood cultures, *Staphylococcus aureus* was identified in 17 (43.6%), *Burkholderia* species in 10 (25.6%) and non‐tuberculous Mycobacterium species in 3 (7.7%). Among the 218 non‐acute hepatitis C infections, 50 (22.9%) were hepatitis C virus (HCV) RNA positive. Overall, HCV RNA was present in 114 of 282 infections (40.4%). The total inflation‐adjusted inpatient cost was $8.39 million, with a median cost per infection of $11,602 (interquartile range, $7426–$34,544).

**Conclusion:**

Injection‐related infections among incarcerated people who inject drugs were associated with substantial morbidity and healthcare costs in this large hospital cohort. A wide clinical spectrum was observed, including atypical pathogens, and clinically overt acute hepatitis C requiring hospital admission. These findings describe a significant burden of preventable disease in custodial settings and support the introduction of established primary prevention and harm‐reduction interventions in prisons.

## Plain Language Summary

**The known:** Injection‐related infections drive preventable hospitalisations among people who inject drugs, with prisons amplifying risk through unsafe injecting conditions.

**The new:** To our knowledge, this is the first Australian study to characterise the clinical spectrum, microbiology and hospital costs of injection‐related infections among incarcerated people who inject drugs, identifying high rates of acute hepatitis C, atypical pathogens and $8.39 million in hospital costs.

**The implications:** Prisons are a critical setting for reform. Implementing evidence‐based harm reduction—including expanded opioid agonist therapy and prison‐based needle‐syringe programs—offers a potential cost‐saving opportunity to prevent injection‐related infections, reduce hepatitis C transmission and close a long‐standing public health gap.

## 1 Introduction

People who inject drugs are at significantly higher risk of injection‐related infections and often require hospitalisation [1]. Common infections include skin and soft tissue infections, bacteraemia, infective endocarditis and osteomyelitis, as well as blood‐borne viruses such as hepatitis B virus (HBV) and hepatitis C virus (HCV). Injection‐related infections are associated with unsafe injecting practices and limited access to sterile equipment [2]. Increased hospitalisation rates for people who inject drugs with bacterial and blood‐borne virus infections place considerable strain on healthcare resources [3, 4, 5, 6] and are associated with sub‐optimal treatment and a high rate of readmission from reinfection [7, 8, 9].

Incarcerated individuals face an even greater burden of injection‐related infections. In Australia, it has been reported that up to 65% of prisoners have injected drugs while in prison [10]. Risk for injection‐related infections through unsafe injection is compounded in prisons [11]. The higher prevalence of blood‐borne viruses in the prison population, especially HCV, increases the likelihood of transmission [12, 13].

Descriptive data on serious infections requiring hospitalisation among incarcerated Australians are scarce, despite reports of infectious disease outbreaks in prisons [14]. This study addresses this gap by describing the clinical spectrum, microbiology and hospital costs of injection‐related infections in a large cohort of incarcerated people who inject drugs.

## 2 Methods

### 2.1 Study Design and Setting

This was a retrospective cohort study, reported in accordance with the Strengthening the Reporting of Observational Studies in Epidemiology (STROBE) statement (Supporting Information, Section S1), of incarcerated patients who presented with an injection‐related infection to the Princess Alexandra Hospital, Brisbane, Australia, between 1 July 2019 and 30 June 2023 [15]. The Princess Alexandra Hospital is a metropolitan quaternary‐level teaching hospital with 1038 beds and a catchment of more than 1.6 million people. Within the hospital, a specialised 12‐bed maximum security unit provides acute inpatient care for individuals in Queensland Corrective Services custody. This secure unit is the primary secure hospital facility for South‐East Queensland, serving about 60% of the state's incarcerated patients. The mean daily prisoner population in Queensland during the study period was 8883 (2019–2020), 9476 (2020–2021), 9589 (2021–22) and 9865 (2022–2023), corresponding to 37,813 prisoner‐years of custodial exposure across the 4‐year period [16].

### 2.2 Participants

Incarcerated subjects admitted to the Princess Alexandra Hospital were identified from discharge summaries through a search of the electronic medical record. Using a range of International Classification of Diseases 10 codes, admissions related to injection‐related infections and injecting drug use were ascertained (Supporting Information, Section S2). We defined injection‐related infections to include the following: soft tissue infections, such as abscesses, cellulitis or thrombophlebitis; bloodstream infections; infective endocarditis; vertebral osteomyelitis and/or epidural abscesses; osteomyelitis; septic arthritis; respiratory infections; deep abscesses; central nervous system infections; blood‐borne viruses such as HBV, acute and chronic HCV, as defined by the European Association for the Study of the Liver (EASL) and human immunodeficiency virus (HIV); and other infections likely associated with injecting drug use [17].

Medical notes were reviewed for all individuals. Patients were included in the study if they were admitted to hospital with an injection‐related infection and had injected within the last 6 months as the documented route of infection. Patient databases within the Princess Alexandra Hospital Alcohol and Drug Assessment Unit and the Infectious Diseases Unit were also cross‐referenced. For patients with multiple admissions during the study period, each admission was recorded. Although data were collected at the level of individual hospital admissions, we identified and linked multiple admissions related to the same infection (infection‐level data).

### 2.3 Data Sources and Collection

Following review of medical records by study investigators (AP, MC, JY and CS), data for all eligible cases were extracted into a single formalised electronic case report form from the Queensland Health integrated electronic medical record, which is linked across public hospitals in the state. Pathology results are also linked statewide, allowing for cross‐referencing. Microbiological data, including blood culture and site‐specific culture results, were obtained directly from the pathology laboratory information system and linked to individual admissions. Data collected included patient demographics, admission characteristics (length of stay, treating team, infectious diseases consultation and diagnoses), laboratory and microbiological findings including blood‐borne virus screening, substance use history, opioid agonist therapy (OAT) status and relevant medical and psychiatric comorbidities. In this study, sex (male/female) was defined as biological sex recorded in the medical record. First Nations status and ethnicity were obtained from routinely collected hospital administrative data, based on self‐identification recorded in the medical record. Infection severity was assessed by requirement for surgical or radiological source control.

The primary outcomes were infection type, microbiological findings, requirement for surgical or radiological source control, hospital length of stay and inpatient healthcare cost. Secondary descriptive outcomes included infectious diseases consultation, patient‐initiated discharge against medical advice, HCV status and crude annualised rates of hospital‐treated infection per 1000 prisoner‐years. Admissions were matched to actual inpatient healthcare costs using Australian Refined Diagnosis‐Related Groups and Activity‐Based Costing methods, with costs adjusted for inflation to 1 January 2025; correctional system costs were not included [18].

All data was recorded in a pre‐designed Excel spreadsheet, incorporating drop‐down menus, where applicable for standardisation and stored on a password‐protected hospital computer.

### 2.4 Analysis

All analyses were carried out using SPSS 29.0. Descriptive analyses were expressed as frequency (percentages, %), mean (standard deviation, SD) and median (interquartile range, IQR) depending on data distribution. Crude annualised rates of hospital‐treated infection were calculated per 1000 prisoner‐years using the Queensland mean daily prisoner population as the denominator.

### 2.5 Ethics

The Human Research Ethics Committee (HREC) Sub‐Committee of the Princess Alexandra Hospital (HREC Reference: EX/2023/QMS/100794) granted an ethics waiver, the study protocol having been assessed in accordance with the National Health and Medical Research Council's Ethical Considerations in Quality Assurance and Evaluation Activities.

## 3 Results

### 3.1 Demographics

A total of 265 patients accounted for 282 unique infections and a total of 321 hospital admissions during the study period. Across 37,813 prisoner‐years of custodial exposure, this corresponded to crude annualised rates of 7.5 injection‐related infections and 8.5 hospital admissions per 1000 prisoner‐years. Excluding acute hepatitis C, the crude annualised rate of bacterial injection‐related infections was 5.8 per 1000 prisoner‐years.

The mean age was 33 years (SD, 7.4 years), and 241 (90.9%) were male. There were 247 patients (93.2%) that were born in Australia, with 76 (28.7%) identified as First Nations. Substance use before incarceration was documented in 230 (81.6%) infections, with opioids (49.1%) and amphetamines (42.6%) being the most reported preferred primary drug of use. A history of OAT before incarceration was documented in 65 (23.0%) infections. None of the patients admitted with injection‐related infections were registered on an OAT program at the time of admission. In 188 infection episodes (66.7%), the person had a documented history of HCV infection, and 59 infection episodes (20.9%) occurred in people who had previously been admitted to hospital with an injection‐related infection. The median length of stay was 5 days (IQR, 3–15 days), with a mean of 12 days (SD, 16 days). Infectious disease consultation occurred in 130 (46.1%) injection‐related infections. Patient‐initiated discharge against medical advice occurred in 34 (10.6%) of admissions. There were no hospital deaths. Table 1 summarises the characteristics of the cohort.

**TABLE 1:** Characteristics of cohort.

Characteristic	Value
Number of admissions	321
Number of patients	265
Number of unique injection‐related infections	282
Age, in years (mean, SD)	33 (7.4)
Sex (% male)	241 (90.9)
Born in Australia (%)	247 (93.2)
First Nations (%)	76 (28.7)
Reported substance use before incarceration (%)	230 (81.6)
Primary drug of use among those with documented substance use: opioids (%)	113 (49.1)
Primary drug of use among those with documented substance use: methamphetamine (%)	98 (42.6)
Community history of opioid agonist therapy (%)	65 (23.0)
Reported history of HCV (%)	188 (66.7)
Previous hospitalisation with injection‐related infections (%)	59 (20.9)
Patient‐initiated discharge (%)	34 (10.6)
Median (IQR) length of stay (days)	5 (3–15)
Mean (SD) length of stay (days)	12 (16)

*Note:* Data are number (%) unless otherwise indicated. Percentages are based on the number of unique injection‐related infections (*n* = 282), unless otherwise specified. Percentages for primary drug of use are based on infections with documented substance use before incarceration (*n* = 230). Age and length of stay are reported as mean (SD) or median (IQR), as appropriate.

Abbreviations: HCV, hepatitis C virus; IQR, interquartile range; SD, standard deviation.

### 3.2 Medical and Psychiatric Comorbidities

Psychiatric illness was documented in 136 infections (48.2%). The most frequent diagnoses were depression (58/282, 20.6%), anxiety (28/282, 9.9%) and schizophrenia (18/282, 6.4%); other recorded psychiatric conditions (32/282, 11.3%) included bipolar affective disorder, post‐traumatic stress disorder, antisocial personality disorder, attention deficit disorder and borderline personality disorder.

Of the 282 infections, 199 (70.6%) had no documented medical comorbidities, 21 (7.4%) reported a history of asthma, 19 (6.7%) chronic pain, 11 (3.9%) type 2 diabetes mellitus, 3 (1.1%) chronic obstructive pulmonary disease and 2 (0.7%) ischaemic heart disease.

### 3.3 Injection‐Related Infections

Of the 282 infections, 77 (27.3%) infections were a soft tissue infection, 64 (22.7%) acute HCV infection and 43 (15.2%) cellulitis. For musculoskeletal infections, 14 (5.0%) were sacroiliac joint septic arthritis, 2 (0.7%) lower limb septic arthritis, 9 (3.2%) other joint septic arthritis, 11 (3.9%) discitis, 8 (2.8%) vertebral osteomyelitis and 7 (2.5%) other osteomyelitis. Infective endocarditis accounted for 13 (4.6%) injection‐related infections, bacteraemia without a defined focus for 8 (2.8%), and 26 (9.2%) were attributed to other infections. Surgical or radiological source control was required in 95 (33.7%) infections.

Forty‐four blood stream infections were identified from cultures, of which five were deemed contaminants. Of the remaining 39 true‐positive blood stream infections, *Staphylococcus aureus* was identified in 17 (43.6%; 13 methicillin‐sensitive *S. aureus* [MSSA], 4 methicillin‐resistant *S. aureus* [MRSA]), *Burkholderia* species in 10 (25.6%), *Enterococcus faecalis* in 3 (7.7%), non‐tuberculous Mycobacterium species in 3 (7.7%), *Klebsiella pneumoniae* in 2 (5.1%) and single isolates of *Serratia marcescens* , *Candida parapsilosis* , *Pseudomonas aeruginosa* and *Streptococcus parasanguinis* . Six blood cultures were polymicrobial.

There were 122 culture‐positive non‐blood specimens identified. *S. aureus* was detected in 71 cases (58.2%), comprising 27 MSSA and 44 MRSA isolates. Other organisms included *P. aeruginosa* (14/122, 11.5%), non‐tuberculous *Mycobacterium* species (7/122, 5.7%), *Burkholderia* species (5/122, 4.1%) and *Streptococcus* species (21/122, 17.2%). Additional organisms were identified less frequently. Polymicrobial infection was present in 24 cases (19.7%), and organism counts are therefore not mutually exclusive.

Across all 166 culture‐positive infection episodes (including contaminants), *Burkholderia* species were identified in 15 episodes (9.0%) and non‐tuberculous *Mycobacterium* species in 10 episodes (6.0%), predominantly *M. abscessus* complex organisms. Detailed microbiology data by pathogen and culture are available in Supporting Information, Section S3, Tables S1–S4.

Acute HCV infection was the primary diagnosis in 64 (22.7%) injection‐related infections, corresponding to a crude annualised rate of 1.7 hospitalised acute HCV cases per 1000 prisoner‐years. Among admissions for acute HCV, median alanine aminotransferase concentration was 1290 U/L (IQR, 772–2220 U/L) and median bilirubin concentration was 102 μmol/L (IQR, 67–141 μmol/L), and median International Normalized Ratio (INR) was 1.1 (IQR, 1.0–1.3), consistent with clinically apparent, acute icteric hepatitis. Among the 218 non‐HCV admissions, 50 (22.9%) had detectable HCV RNA, 30 (13.8%) were HCV RNA negative and in 138 (63.3%), HCV RNA testing was not performed. Overall, HCV RNA was present in 114 of 282 infections (40.4%).

### 3.4 Cost to the Healthcare System

Costing data were available for 279 of 282 infections. Adjusted for inflation, the total inpatient cost over the study period was $8.39 million. The mean cost per infection was $30,089 (SD, $39,926), with a median of $11,602 (IQR, $7426–$34,544), reflecting a highly skewed distribution. A quarter of admissions accounted for 71.5% of total expenditure. Median costs varied substantially by diagnosis. Table 2 summarises costs, infection counts and length of stay by diagnosis.

**TABLE 2:** Number of cases, length of stay and cost by principal diagnosis.

Principal diagnosis	Number (%)	Median length of stay, days (IQR)	Median cost, $ (IQR)
Discitis	11 (3.9%)	16 (15–46)	62,237 (58,360–116,502)
Vertebral osteomyelitis	8 (2.8%)	27 (17–40)	89,094 (65,305–145,528)
Other bone osteomyelitis	7 (2.5%)	15 (11–22)	24,894 (18,498–55,448)
Sacroiliac joint septic arthritis	14 (5.0%)	18 (13–29)	62,522 (40,589–93,654)
Lower limb septic arthritis	2 (0.7%)	31 (4–57)	79,200 (13,968–144,433)
Other joint septic arthritis	9 (3.2%)	14 (12–16)	30,310 (24,957–42,952)
Infective endocarditis	13 (4.6%)	34 (22–42)	67,696 (45,474–125,291)
Soft tissue infection	77 (27.3%)	4 (2–7)	9398 (6704–14,131)
Bacteraemia without focus	8 (2.8%)	15 (7–26)	22,681 (16,119–62,691)
Cellulitis	43 (15.2%)	4 (3–6)	7426 (4784–10,874)
Acute hepatitis C	64 (22.7%)	4 (3–6)	10,418 (6854–14,737)
Other	26 (9.2%)	7 (3–22)	22,751 (7248–63,725)

*Note:* Data are number (%) unless otherwise indicated. Percentages are based on the total number of unique injection‐related infections (*n* = 282). Costs are inflation‐adjusted to 1 January 2025. Costing data were available for 279 of 282 infections.

Abbreviation: IQR, interquartile range.

## 4 Discussion

To our knowledge, this is the first Australian study to comprehensively describe hospitalised injection‐related infections among incarcerated people who inject drugs. By analysing a large real‐world cohort over 4 years, we report both the scale and clinical complexity of injection‐related infections in prisons and their substantial, yet potentially preventable, financial burden on the public hospital system.

Our cohort was largely young, male, Australian‐born and socio‐economically marginalised, with high rates of substance use and psychiatric illness. First Nations people were overrepresented, reflecting disproportionate incarceration rates and unmet health needs [19, 20]. Soft tissue infections and musculoskeletal infections were the leading causes of hospitalisation, alongside acute HCV infection. By contrast, bloodstream infections and deep‐seated infections were less frequent but required intensive healthcare resources. Nearly half of all cases required specialist infectious disease input, and one‐third required surgical or radiological source control, underscoring their complexity.

The crude annualised rates reflect hospital‐admitted infection relative to custodial exposure and are likely conservative. The denominator included the entire custodial population rather than only people who inject drugs and only infections requiring hospital admission. Cases were derived from a single secure inpatient unit serving about 60% of the Queensland prison population, without catchment adjustment. The true statewide burden is therefore likely higher than the estimate suggests.

Patient‐initiated discharge against medical advice occurred in 34 admissions (10.6%). Although this may appear counterintuitive in a custodial population, it is a recognised occurrence and likely reflects challenges similar to those seen in community settings, including untreated drug dependence, as well as custodial, security or court‐related processes that may interrupt inpatient treatment. This is relevant to severe bacterial and blood‐borne viral infections, for which treatment interruption may contribute to incomplete therapy, recurrence and avoidable readmissions.

Notably, none of the individuals hospitalised with injection‐related infections were enrolled in OAT at admission. While causal inference is not possible, this observation is consistent with the established protective effect of OAT [21]. During much of the study period, custodial OAT provision in Queensland was limited. National pharmacotherapy surveillance data demonstrate minimal prison‐based dosing before 2022, and fewer than 0.6% of admissions in our cohort originated from facilities involved in Phase 1 implementation of long‐acting injectable buprenorphine [22]. Statewide rollout continued to progress during this period and was still described as ongoing in official reports in 2023–2024 [23]. Long‐acting injectable buprenorphine represents an important custodial harm‐reduction strategy given its sustained dosing and reduced diversion risk [24]. Experience from New South Wales demonstrates that large‐scale prison OAT implementation is feasible and safe [25].

A novel microbial finding was the culture of atypical environmental pathogens, *Burkholderia* spp. and non‐tuberculous *Mycobacterium* spp. Their incidence was far higher than would be expected in typical community‐acquired infections, with no evidence of similar infections in community‐dwelling people who inject drugs admitted to our hospital with infection in the same period [26, 27, 28]. These organisms are intrinsically multidrug‐resistant. Their emergence in our cohort may reflect the high‐risk injecting micro‐environment and delayed access to medical care at the early stages of infection. Our findings contribute new evidence to the literature on atypical injection‐related infections in incarcerated populations, highlighting an issue that has been largely overlooked in Australian and global public health research [29, 30, 31].

The higher proportion of MRSA in non‐blood culture isolates compared with bloodstream isolates is consistent with local epidemiology [32, 33]. In Queensland, community‐associated ST93 MRSA predominates in skin and soft tissue infections, whereas invasive bloodstream infections are more commonly caused by MSSA. In the absence of temporal clustering or molecular typing, these findings do not support inference of outbreak‐type transmission.

While prison HCV literature has focused primarily on prevalence, testing uptake and treatment outcomes, hospitalised clinically apparent acute HCV has been rarely described. Despite prioritising incarcerated populations for HCV treatment and the availability of direct‐acting antivirals, acute HCV admissions observed in this cohort reflected clinically overt disease requiring hospitalisation, rather than asymptomatic infections detected through routine screening. Most HCV infections (50 of 64) were managed under hepatology services in a tertiary referral centre, with the remainder admitted under infectious diseases or general medicine and reviewed by hepatology services, supporting diagnostic certainty. The high occurrence suggests ongoing transmission within prisons and challenges in early detection in high‐risk environments.

Moreover, acute icteric HCV likely represents only a subset of incident infections. Most acute infections remain asymptomatic and may not be detected between testing episodes, meaning that hospitalised cases represent the visible component of a larger incident burden. In this context, acute HCV admissions can function as sentinel events, signalling persistent transmission and ongoing challenges to prevention, surveillance and continuity of care within prisons [13, 34, 35]. The parallel emergence of atypical environmental pathogens alongside acute icteric HCV suggests shared upstream determinants related to high‐risk injecting conditions within prison environments. While prison blood‐borne virus programs have been central to HCV elimination efforts, the often‐cyclical nature of testing and treatment delivery, characterised by periods of scale‐up followed by attrition, may be insufficient to fully suppress transmission in high‐risk custodial settings [36]. Test‐and‐treat strategies alone are unlikely to eliminate infection without complementary prevention measures and effective treatment of substance dependence [13, 35]. Without structural interventions, progress towards national hepatitis C elimination targets may be vulnerable, while preventable hospitalisations and downstream costs continue to accrue [37, 38].

The persistence of these preventable infections also reflects Australia's long‐standing lack of access to needle‐syringe programs (NSPs) in prisons [39]. Community‐based NSPs have significantly reduced community‐based injection‐related harms; however, Australia has yet to establish such a national program or policy for prisons [40]. Several countries have introduced prison‐based NSPs with demonstrable reductions in HIV and HCV transmission, and without increases in drug use or security incidents, demonstrating their effectiveness and safety [41, 42, 43]. The absence of this evidence‐based harm‐reduction measure in Australian prisons represents a notable gap in the national response to injection‐related harms [44, 45]. Incarceration should not be a barrier to evidence‐based prevention; yet, currently, people who inject drugs in custody are unable to access interventions that are standard in the community (Mandela rules), exacerbating health inequities between prison and community settings [46].

Our findings highlight the substantial financial burden associated with these infections. Over the 4‐year study period, injection‐related infections accrued a total inpatient cost of $8.39 million. This figure underestimates the true economic impact, as it excludes prisoner transport and security escorts, outpatient care, HCV treatment, long‐term disability and costs associated with recurrent admissions. Given the observed size of inpatient expenditure, preventive interventions that reduce infection incidence would be expected to generate downstream cost savings within the health system.

Recent modelling estimated that a national prison‐based NSP could avert about 17% of injection‐related infection hospitalisations over 5 years, yielding a projected return of $2.60 for every $1 invested [47]. However, these models assumed a mean hospitalisation cost of $13,000. In contrast, our real‐world cohort incurred a mean cost of $30,089 per infection, suggesting that the return on investment for custodial prevention may be closer to that of established community‐based harm‐reduction programs than current models assume [48, 49].

### 4.1 Strengths

We employed comprehensive case‐finding across multiple data sources, improving capture of all eligible cases and minimising selection bias. We obtained detailed microbiological data, which enabled us to identify atypical infections and matched this with actual hospital costs to ensure accurate hospital cost estimation. The study was conducted at Queensland's primary hospital for prisoner health, housing the state's only secure inpatient unit for prisoners. This setting gives confidence that the study population is representative of the incarcerated population in Queensland.

### 4.2 Limitations

As a retrospective study, we relied on the accuracy and completeness of medical record documentation. Regional variation in pathogen epidemiology may not be fully reflected in this single‐centre cohort, and differing models of custodial healthcare delivery may limit the generalisability of findings to other Australian jurisdictions. Detailed injecting behaviours, including frequency and practices during incarceration, were inconsistently documented and could not be reliably analysed. We acknowledge that some infections may have been acquired before incarceration. We also did not capture infections managed solely within correctional health services or those treated in the emergency department, as our analysis was intentionally focused on severe cases requiring hospital admission. As this was a retrospective study, we were unable to collect qualitative data, which may have provided additional context for interpreting these findings. However, recent qualitative research conducted in Queensland prisons has described structural and social conditions that foster equipment scarcity, sharing practices and delayed access to healthcare, providing contextual insight consistent with the infection patterns observed in our cohort [50]. Prospective mixed‐methods research integrating clinical, microbiological and lived‐experience data represents an important area for future work [51].

## 5 Conclusions

In conclusion, we describe a substantial burden of hospital‐admitted injection‐related infections among incarcerated people who inject drugs, including clinically significant acute HCV, severe bacterial infections, atypical environmental pathogens and high associated inpatient healthcare costs. Importantly, these infections arise from upstream and largely preventable conditions. In the absence of sustained investment in harm reduction and evidence‐based treatment for drug dependence within custodial settings, it is likely that hospital services will continue to bear the downstream burden of potentially preventable infection.

## Author Contributions

Andrew Palmer and Paul J. Clark: conceptualisation. Andrew Palmer, Matthew Carter, Jeremy Yeo and Cecilia Shim: data curation. Matthew Carter: investigation and formal analysis. Naomi Moy, Naomi Runnegar and Elliott G. Playford: formal analysis. Andrew Palmer: writing – original draft. All authors: review and editing.

## Funding

The authors acknowledge the financial support provided to Andrew Palmer through the Royal Australasian College of Physicians Research Entry Scholarship. The Royal Australasian College of Physicians had no role in the in‐study design, data collection, analysis or interpretation, reporting or publication.

## Disclosure

Not commissioned; externally peer reviewed.

## Conflicts of Interest

Andrew Palmer has provided an educational presentation and participated in an advisory board for AbbVie related to hepatitis C (including Maviret and acute HCV management) within the past 36 months. No personal payments were received. All honoraria were directed to the Princess Alexandra Hospital Foundation.

## Supporting information


**Data S1:** Supporting Information and tables.

## Data Availability

All data/code/materials relevant to the study are included in the article or are available in the Supporting Information file.
