# Supplementary material for: Hospital‐Admitted Injection‐Related Infections Among Incarcerated People Who Inject Drugs in Australia: A Retrospective Cohort Study
Source: Med J Aust. 2026 Jun 14;224(6):e70224. doi: 10.5694/mja2.70224 (PMC13265619; doi:10.5694/mja2.70224)
Supplement: Supplementary file 1 — Data S1: Supporting Information and tables. [file MJA2-224-0-s001.pdf]

Supporting Information

Supplementary material

This appendix was part of the submitted manuscript and has been peer reviewed.
It is posted as supplied by the authors.

Appendix to: Palmer A, Carter M, Yeo J, et al. Hospital-admitted injection-related infections among incarcerated people who inject drugs in Australia: a retrospective cohort study. *Med J Aust* 2026; doi: 10.5694/mja2.70224

Section S1

STROBE Statement for: "Injection-related infections among incarcerated Australians who inject drugs: clinical spectrum, microbiology, virology and healthcare costs in a real-world hospital cohort."

	Item No	Recommendation	Page in manuscript
**Title and abstract**	1	(*a*) Indicate the study's design with a commonly used term in the title or the abstract	1
		(*b*) Provide in the abstract an informative and balanced summary of what was done and what was found	2-3
**Introduction**			
Background/rationale	2	Explain the scientific background and rationale for the investigation being reported	5
Objectives	3	State specific objectives, including any prespecified hypotheses	5
**Methods**			
Study design	4	Present key elements of study design early in the paper	6
Setting	5	Describe the setting, locations, and relevant dates, including periods of recruitment, exposure, follow-up, and data collection	6
Participants	6	(*a*) Give the eligibility criteria, and the sources and methods of selection of participants. Describe methods of follow-up	6-7
		(*b*) For matched studies, give matching criteria and number of exposed and unexposed	NA
Variables	7	Clearly define all outcomes, exposures, predictors, potential confounders, and effect modifiers. Give diagnostic criteria, if applicable	NA
Data sources/ measurement	8*	For each variable of interest, give sources of data and details of methods of assessment (measurement). Describe comparability of assessment methods if there is more than one group	7-8
Bias	9	Describe any efforts to address potential sources of bias	NA
Study size	10	Explain how the study size was arrived at	NA
Quantitative variables	11	Explain how quantitative variables were handled in the analyses. If applicable, describe which groupings were chosen and why	8
Statistical methods	12	(*a*) Describe all statistical methods, including those used to control for confounding	8
		(*b*) Describe any methods used to examine subgroups and interactions	NA
		(*c*) Explain how missing data were addressed	NA
		(*d*) If applicable, explain how loss to follow-up was addressed	NA
		(*e*) Describe any sensitivity analyses	NA
**Results**			
Participants	13*	(a) Report numbers of individuals at each stage of study—eg numbers potentially eligible, examined for eligibility, confirmed eligible, included in the study, completing follow-up, and analysed	9
		(b) Give reasons for non-participation at each stage	NA

		(c) Consider use of a flow diagram	NA
Descriptive data	14*	(a) Give characteristics of study participants (eg demographic, clinical, social) and information on exposures and potential confounders	9
		(b) Indicate number of participants with missing data for each variable of interest	10
		(c) Summarise follow-up time (eg, average and total amount)	
Outcome data	15*	Report numbers of outcome events or summary measures over time	9
Main results	16	(*a*) Give unadjusted estimates and, if applicable, confounder-adjusted estimates and their precision (eg, 95% confidence interval). Make clear which confounders were adjusted for and why they were included	10,11,12
		(*b*) Report category boundaries when continuous variables were categorized	NA
		(*c*) If relevant, consider translating estimates of relative risk into absolute risk for a meaningful time period	NA
Other analyses	17	Report other analyses done—eg analyses of subgroups and interactions, and sensitivity analyses	NA
**Discussion**			
Key results	18	Summarise key results with reference to study objectives	13
Limitations	19	Discuss limitations of the study, taking into account sources of potential bias or imprecision. Discuss both direction and magnitude of any potential bias	17
Interpretation	20	Give a cautious overall interpretation of results considering objectives, limitations, multiplicity of analyses, results from similar studies, and other relevant evidence	18
Generalisability	21	Discuss the generalisability (external validity) of the study results	17
**Other information**			
Funding	22	Give the source of funding and the role of the funders for the present study and, if applicable, for the original study on which the present article is based	NA

*Give information separately for exposed and unexposed groups.

Note: An Explanation and Elaboration article discusses each checklist item and gives methodological background and published examples of transparent reporting. The STROBE checklist is best used in conjunction with this article (freely available on the Web sites of PLoS Medicine at http://www.plosmedicine.org/, Annals of Internal Medicine at http://www.annals.org/, and Epidemiology at http://www.epidem.com/). Information on the STROBE Initiative is available at http://www.strobe-statement.org.

Section S2

Broad International Classification of Diseases 10 (ICD-10-AM) chapter ranges and diagnostic codes were used for initial electronic case identification to maximise sensitivity. These are listed below. Records were subsequently restricted to admissions occurring within the secure unit, and all retrieved medical records were manually reviewed to confirm eligibility according to predefined clinical criteria for injecting-related infection.

Certain infectious and parasitic diseases
A00–B99
Sepsis and bacterial infections
A40.0–A40.9
A41.0–A41.4, A41.50–A41.52, A41.58, A41.8–A41.9
A49.00–A49.01, A49.11–A49.12, A49.2–A49.3, A49.81–A49.89, A49.9
Viral hepatitis and blood-borne viruses
B15.0–B15.9
B16.0–B16.2, B16.9
B17.0–B17.2, B17.8–B17.9
B18.0–B18.2, B18.8–B18.9
B19.0, B19.9
B20–B24
Mental and behavioural disorders due to psychoactive substance use
F10–F19
Opioids
F11.0–F11.9
Cannabinoids
F12.0–F12.9
Sedatives / hypnotics
F13.00–F13.01, F13.09
F13.10–F13.11, F13.19
F13.20–F13.21, F13.29
F13.30–F13.31, F13.39
F13.40–F13.41, F13.49
F13.50–F13.51, F13.59
F13.60–F13.61, F13.69
F13.70–F13.71, F13.79
F13.80–F13.81, F13.89
F13.90–F13.91, F13.99
Cocaine
F14.0–F14.9
Other stimulants
F15.00–F15.02, F15.09
F15.10–F15.12, F15.19
F15.20–F15.22, F15.29
F15.30–F15.32, F15.39
F15.40–F15.42, F15.49
F15.50–F15.52, F15.59
F15.60–F15.62, F15.69
F15.70–F15.72, F15.79
F15.80–F15.82, F15.89
F15.90–F15.92, F15.99
Hallucinogens
F16.00–F16.01, F16.09

F16.10–F16.11, F16.19
F16.20–F16.21, F16.29
F16.30–F16.31, F16.39
F16.40–F16.41, F16.49
F16.50–F16.51, F16.59
F16.60–F16.61, F16.69
F16.70–F16.71, F16.79
F16.80–F16.81, F16.89
F16.90–F16.91, F16.99
Tobacco
F17.1–F17.9
Volatile solvents
F18.0–F18.9
Multiple/other substances
F19.1–F19.9
Diseases of the nervous system
G00–G99
G06.0–G06.2
Diseases of the circulatory system
I00–I99:
I33.0
Diseases of the skin and subcutaneous tissue
L00–L99
Cutaneous abscess
L02.0–L02.3
L02.40–L02.43
L02.8–L02.9
Cellulitis
L03.01–L03.02
L03.12–L03.14, L03.19
L03.2–L03.3
L03.8–L03.9
Musculoskeletal system and connective tissue
M00–M99
Septic arthritis
M00.00–M00.29
M00.80–M00.89
M00.90–M00.99
Infective myositis
M60.01–M60.09
Pregnancy, childbirth and puerperium
O00–O99
O85
Injury, poisoning and other consequences of external causes
S00–T98
T79.3
T89.01–T89.02
External causes
W46
Z codes
Z21

Section S3

Table S1. Positive Blood Cultures by Environmental Niche

**Environmental Niche**	**Organism**	**Number**
	Contaminant	5
**Skin Flora**	*Staphylococcus aureus* (MSSA)	13
	Staphylococcus aureus (MRSA)	4
**GI Flora**	*Klebsiella pneumoniae*	2
**Oral or GI Flora**	*Enterococcus faecalis*	3
	Streptococcus parasanguinis	1
**Environmental Flora**	*Burkholderia* species (total)	10
	B. cenocepacia rec A clade B	8
	B. gladioli	1
	B. cepacia	1
	Pseudomonas aeruginosa	1
**Environmental Flora (Acid-fast bacilli)**	Non-tuberculous *Mycobacterium* species (total)	3
	Mycobacterium abscessus	2
	Mycobacterium fortuitum	1
**Skin, GI or Oral Flora**	*Candida parapsilosis*	1
**Environmental or GI Flora**	*Serratia marcescens*	1
	Polymicrobial cultures	6

Table S2: Positive Blood Cultures by Gram Classification

**Gram Classification**	**Organism**	**Number**
	Contaminant	5
**Gram Positive (Cocci)**	*Staphylococcus aureus* (MSSA)	13
	Staphylococcus aureus (MRSA)	4
	Enterococcus faecalis	3
	Streptococcus parasanguinis	1
**Gram Negative (Bacilli)**	*Burkholderia species* (total)	10
	B. cenocepacia rec A clade B	8
	B. gladioli	1
	B. cepacia	1
	Pseudomonas aeruginosa	1
	Klebsiella pneumoniae	2
	Serratia marcescens	1
**Acid-Fast Bacilli**	*Non-tuberculous Mycobacterium species (total)*	3
	Mycobacterium abscessus	2
	Mycobacterium fortuitum	1
**Yeast**	*Candida parapsilosis*	1
	Polymicrobial cultures	6

Table S3: Positive Non-Blood Cultures by Environmental Niche

Environmental Niche	Organism	Number
**Skin Flora**	*Staphylococcus aureus* (MSSA)	27
	Staphylococcus aureus (MRSA)	44
	Streptococcus species	21
	Cultibacterium acnes	3
**GI Flora**	*Klebsiella pneumoniae*	3
	Mixed enteric flora	1
**Environmental Flora**	*Burkholderia* species	5
	Non-tuberculous *Mycobacterium species*	7
	Pseudomonas aeruginosa	14
**Environmental or GI Flora**	*Serratia marcescens*	4
	Enterococcus faecalis	1
**Skin, GI or Environmental Flora**	*Candida* species	4
—	Polymicrobial specimens	24

Table S4: Positive Non-Blood Cultures by Gram Classification

Gram Classification	Organism	Number
**Gram Positive (Cocci)**	*Staphylococcus aureus* (MSSA)	27
	*Staphylococcus aureus (*MRSA)	44
	Streptococcus species	21
	Enterococcus faecalis	1
**Gram Positive (Bacilli)**	*Cultibacterium acnes*	3
**Gram Negative (Enterobacterales)**	*Klebsiella pneumoniae*	3
	Serratia marcescens	4
**Gram Negative (Non-fermenting)**	*Burkholderia* species	5
	Pseudomonas aeruginosa	14
**Acid-Fast Bacilli**	Non-tuberculous *Mycobacterium* species	7
**Yeast**	*Candida* species	4
	Mixed enteric flora	1
	Polymicrobial specimens	24
